# Integrative Machine Learning and Experimental Validation Identify MYBL2 as a Prognostic Biomarker and Therapeutic Target in Hepatocellular Carcinoma

**DOI:** 10.32604/or.2026.075284

**Published:** 2026-04-22

**Authors:** Ya-Ling Yang, Ying-Hsien Huang, Hung-Yu Lin

**Affiliations:** 1 Department of Anesthesiology, Kaohsiung Chang Gung Memorial Hospital and Chang Gung University College of Medicine, Kaohsiung, Taiwan; 2 Department of Pediatrics, Kaohsiung Chang Gung Memorial Hospital and Chang Gung University College of Medicine, Kaohsiung, Taiwan; 3 Department of Post-Baccalaureate Medicine, College of Medicine, National Chung Hsing University, Taichung, Taiwan; 4 Research Assistant Center, Show Chwan Memorial Hospital, Changhua, Taiwan

**Keywords:** Hepatocellular carcinoma, MYBL2, biomarker, prognosis, precision medicine, miR-29a, tumor microenvironment

## Abstract

**Background:**

Hepatocellular carcinoma (HCC) presents with poor treatment outcomes, creating an urgent need for novel biomarkers to improve diagnosis, prognosis, and precision medicine. While the MYB family of oncogenes is implicated in cancer, the role and regulatory mechanisms of its member, particularly MYB proto-oncogene like 2 (MYBL2), remain underexplored in HCC. Therefore, this study aimed to systematically validate the clinical significance of MYBL2, elucidate its functional role in tumor progression and drug sensitivity, and identify its upstream regulatory mechanisms using an integrative machine learning and experimental framework.

**Methods:**

We applied an integrative pipeline combining LASSO-based feature selection on TCGA and GEO cohorts, single-cell transcriptomics, pharmacogenomic surveys, and CRISPR dependency screens. These computational approaches were complemented by *in vitro* HepG2 assays, luciferase reporter tests, iTRAQ proteomics, and an *in vivo* western diet/CCl_4_ (WD/CCl_4_) HCC model using miR-29a transgenic mice to investigate a putative regulatory axis.

**Results:**

MYBL2 robustly discriminated tumor from normal liver (AUC = 0.968), and high expression was associated with adverse features, including higher grade, microvascular invasion, HBV positivity, nonresponse to TACE, and worse survival. A nomogram combining MYBL2 with AJCC stage improved 1-, 3-, and 5-year AUCs versus stage alone. MYBL2 correlated with proliferative biomarkers (AFP, MKI67, PCNA, BIRC5) and CRISPR knockout inhibited growth in most HCC lines. High MYBL2 expression was associated with greater sensitivity to sorafenib in pharmacogenomic screens and was linked to an immunosuppressive microenvironment and higher MSI. Mechanistically, miR-29a was shown to suppress MYBL2 translation by directly binding to its 3^′^-UTR; this was validated *in vivo*, where miR-29a transgenic mice were protected from WD/CCl_4_-induced HCC, demonstrating reduced tumor burden, MYBL2 expression, and fibrosis. iTRAQ proteomics further confirmed MYBL2 as a top miR-29a–regulated protein.

**Conclusions:**

MYBL2 is a potent diagnostic and prognostic biomarker in HCC that also predicts sorafenib sensitivity. Our findings establish a clear regulatory link where MYBL2 is a direct and functionally important target of the tumor-suppressive miR-29a. This positions MYBL2 as a tractable target for miR-29a-based therapeutic strategies, warranting clinical validation for patient stratification and treatment development in HCC.

## 1 Introduction

Hepatocellular carcinoma (HCC) represents a formidable global health challenge, ranking as a leading cause of cancer-related mortality worldwide [1]. The incidence of HCC is rising, driven by diverse risk factors including chronic viral hepatitis, alcohol consumption, and metabolic syndrome [2]. Despite recent progress in surgical and systemic therapies, the overall prognosis for patients remains poor, largely due to late-stage diagnosis and tumor heterogeneity [3–7]. This challenging clinical landscape highlights a critical need for novel biomarkers that can facilitate early detection, provide accurate prognostic information, and guide the development of personalized treatment strategies to enhance patient outcomes [8].

In recent years, the scientific community has intensified its efforts to identify novel, druggable targets that could pave the way for more effective and personalized treatment strategies [9]. For instance, recent investigations have identified Vav1 as a promising molecular prognostic biomarker, where its high expression correlates with tumor differentiation, recurrence, and unfavorable survival rates in HCC [10]. Among the various molecular pathways under investigation, the MYB family of transcription factors has emerged as a key player in cancer biology [11–14]. The MYB proto-oncogenes, known for their roles in cell proliferation, differentiation, and apoptosis, have been implicated in the pathogenesis of several malignancies [13,15–17]. This family, comprising MYB, MYBL1, and MYB proto-oncogene-like 2 (MYBL2), consists of proteins that are central regulators of essential cellular processes such as cell cycle progression, differentiation, and apoptosis [13]. While their dysregulation is linked to the pathogenesis of various malignancies, their specific roles and clinical utility within the complex molecular landscape of HCC have not been comprehensively elucidated. Among the MYB family members, MYBL2 has garnered particular interest due to its involvement in cell cycle progression and its aberrant expression in various cancers [14,18]; however, its specific role in HCC remains insufficiently characterized.

While MYBL2 dysregulation has been linked to poor outcomes in colorectal and breast cancers, its specific role within the unique metabolic and immune microenvironment of HCC remains underexplored. Furthermore, existing HCC prognostic models rely heavily on clinicopathological features (e.g., American Joint Committee on Cancer [AJCC] stage) and often lack integration with molecular drivers that capture tumor heterogeneity and therapeutic sensitivity. Recent studies have demonstrated the power of large-scale cohort analyses and advanced computational models in refining cancer prognosis. For instance, nationwide cohort studies have successfully elucidated the impact of concurrent medications like statins on HCC outcomes [19], while innovative ensemble learning frameworks have shown superior performance in risk prediction for complex diseases [20]. Similarly, integrative bioinformatics analyses utilizing gene expression datasets and protein-protein interaction networks have successfully identified top-ranked hub genes as potential therapeutic targets, validating the utility of systems biology in deciphering HCC pathogenesis [21]. Building on these methodological advancements, we applied an integrative machine learning pipeline with a relevant western diet/carbon tetrachloride (WD/CCl_4_) animal model to screen a network of MYB-associated genes and identify robust prognostic candidates. This unbiased computational approach pinpointed MYBL2 as the top candidate with significant diagnostic and prognostic potential.

The primary objectives of our research were threefold: first, to validate the clinical significance of MYBL2 as a biomarker for HCC diagnosis and survival prediction; second, to investigate its functional role in HCC progression and its utility as a predictive marker for sensitivity to standard therapies like sorafenib; and third, to uncover its upstream regulatory mechanisms, leading to the identification of the miR-29a-MYBL2 axis as a novel, targetable pathway. By combining bioinformatics, *in vitro* cell-based assays, and *in vivo* mouse models, our work provides a comprehensive evaluation of MYBL2, positioning it as a promising biomarker and therapeutic target to advance precision oncology for HCC.

## 2 Materials and Methods

### 2.1 Bioinformatics Resources and Data Acquisition/Preprocessing

The primary data for this study were obtained from The Cancer Genome Atlas (TCGA) datasets (v36.0, Jan 2024), systematically downloaded from the University of California Santa Cruz (UCSC) Xena platform (https://xena.ucsc.edu/). These datasets encompassed comprehensive genomic, transcriptomic, and clinical information for HCC patients. Independent validation datasets were acquired from the Gene Expression Omnibus (GEO). Additionally, to establish a baseline for normal tissue gene expression, we incorporated data from the Genotype-Tissue Expression (GTEx) Portal, which offers a comprehensive resource of gene expression data from various healthy human tissues. To ensure data quality and comparability, a rigorous preprocessing pipeline was implemented. Initially, the ‘sva’ package in R was employed to normalize the data and mitigate batch effects and technical variabilities across different experimental runs. This step reduced non-biological sources of variation that could confound downstream analyses. Following normalization, missing data points were addressed using the ‘mice’ (Multivariate Imputation by Chained Equations) package in R. This multiple imputation approach ensures the robust handling of missing data. The resulting preprocessed and complete datasets formed the foundation for subsequent machine learning analyses and experimental validations. All data processing and statistical analyses were performed using R (version 4.1.0) and relevant Bioconductor packages.

### 2.2 Identification and Validation of Prognostic Genes

To identify robust prognostic candidates, we initially screened 22 MYB-family related genes and their direct downstream targets. A univariate Cox proportional hazards regression was applied as a pre-selection filter (threshold *p* < 0.05). To address multicollinearity among the remaining significant candidates, we employed the Least Absolute Shrinkage and Selection Operator (LASSO) regression using the ‘glmnet’ R package (v4.1-2). This method effectively selects the most informative features by shrinking coefficients of correlated predictors to zero. The resulting model, and specifically the prognostic value of MYBL2, was subsequently validated externally using independent cohorts from the ICGC (LIRI-JP) and GEO (GSE144269). This methodology ensured that our model remained both powerful and generalizable. The discriminative accuracy of the identified markers was assessed using the area under the receiver operating characteristic (ROC) curve (AUC), calculated with the pROC R package. To evaluate the prognostic value of the identified genes, we conducted Kaplan-Meier survival analysis using an automatic best cutoff algorithm. This analysis was performed using the online tool KM Plotter [22], which integrates gene expression data with clinical outcomes. We investigated the expression pattern of MYBL2 in both normal tissue and tumor tissue using HCCDB, an integrative molecular database specifically designed for HCC research [23]. To ensure the robustness of our findings, we conducted a comprehensive validation of MYBL2 expression across multiple databases. This validation process utilized web-based applications, including BEST (Biomarker Enrichment and Selection Tool) [24] and TNMplot [25], which integrate data from various sources such as The Cancer Genome Atlas (TCGA), and Genotype-Tissue Expression (GTEx).

### 2.3 Development of a Prognostic Scoring Model

To develop a comprehensive prognostic scoring model that integrates MYBL2 expression levels and AJCC stage, we employed a multistep approach using various statistical methods and R packages. A nomogram-based prognostic scoring model integrating MYBL2 expression levels and AJCC stage was developed to predict survival probability in hepatocellular carcinoma patients. The model development and validation process involved several steps and utilized various R packages for statistical analysis. Initially, a Cox proportional hazards model was constructed using the ‘survival’ package in R, with MYBL2 expression and AJCC stage as predictors and overall survival as the outcome. Based on this model, a nomogram was formulated using the ‘rms’ package, providing a visual representation of the prognostic model and allowing for the prediction of 1-year, 3-year, and 5-year survival probabilities.

To evaluate the performance of the scoring model, ROC curve, risk score analysis, and Kaplan-Meier survival analysis were conducted using R packages. ROC curves were generated using the ‘pROC’ package and the area under the curve (AUC) to assess the model’s discriminative ability. The nomogram calculated risk scores for each patient and stratified them into high-risk and low-risk groups using the median risk score as the cut-off point. The ‘survminer’ package was used for risk score visualization. Kaplan-Meier survival analysis was utilized to compare survival outcomes between the high-risk and low-risk groups, with the log-rank test used to assess the statistical significance of the difference between the survival curves. This analysis was conducted using both the ‘survival’ and ‘survminer’ packages.

### 2.4 Correlation of MYBL2 with Known Prognostic Biomarkers for HCC

To validate the prognostic significance of MYBL2 in HCC, a comprehensive correlation analysis was performed using three distinct datasets: TCGA, International Cancer Genome Consortium (ICGC), and the GEO dataset GSE144269. The analysis was conducted using the Integrative HCC Gene Analysis (IHGA) web application, a specialized tool designed for HCC research [26]. MYBL2 expression levels were correlated with a comprehensive panel of established prognostic biomarkers for HCC. This panel included alpha-fetoprotein (AFP), glypican-3 (GPC3), cyclin-dependent kinase 4 (CDK4), proliferating cell nuclear antigen (PCNA), baculoviral IAP repeat-containing protein 5 (BIRC5), aurora kinase A (AURKA), discs large-associated protein 5 (DLGAP5), cyclin-dependent kinase 1 (CDK1), marker of proliferation Ki-67 (MKI67), forkhead box protein M1 (FOXM1), DNA topoisomerase II alpha (TOP2A), centromere protein F (CENPF), cyclin-dependent kinase inhibitor 3 (CDKN3), abnormal spindle microtubule assembly (ASPM), NIMA-related kinase 2 (NEK2), minichromosome maintenance complex component 2 (MCM2), cell division cycle 20 (CDC20), and cyclin B1 (CCNB1). Pearson’s correlation coefficient was calculated to quantify the strength and direction of the relationship between MYBL2 and each biomarker.

### 2.5 Functional Analysis

The biological role of MYBL2 in hepatocellular carcinoma (HCC) cell viability was examined using CRISPR knockout screen datasets, which are publicly available through the Cancer Dependency Map (DepMap) portal [27]. These datasets were originally published by the Broad Institute’s Project Achilles and the Sanger Institute’s SCORE (Sanger’s Catalogue of Somatic Mutations in Cancer) project [28]. The DepMap integrates these large-scale genomic datasets to provide comprehensive insights into cancer cell vulnerabilities. Gene set enrichment analysis (GSEA) for MYBL2 expression in TCGA-LIHC cohort was performed using GENI (Gene ENrichment Identifier), a web-based tool for gene expression analysis [29]. The causative interplay between MYBL2 gene expression levels and cell states at a single cell transcriptomics level that contribute to cellular development trajectory and fate was analyzed with CellTracer [30].

### 2.6 Analysis of Drugs Sensitivity, Microsatellite Instability and Microenvironment

Drug sensitivity assessment was conducted using bulk sample data from three major pharmacogenomic databases: the Genomics of Drug Sensitivity in Cancer (GDSC), the Cancer Therapeutics Response Portal (CTRP), and the Profiling Relative Inhibition Simultaneously in Mixtures (PRISM) [24]. Drug response data and gene expression profiles of cancer cell lines were analyzed to evaluate the association between MYBL2 expression and drug sensitivity.

To investigate the potential role of MYBL2 in immunotherapy response, we examined the correlation of MYBL2 expression with the microsatellite instability (MSI), a predictive biomarker for immune checkpoint inhibitors. These analyses were performed using the ‘TCGAplot’ package in R, which facilitates the exploration and visualization of TCGA data [31].

The correlation between MYBL2 expression and various components of the tumor-immune microenvironment was comprehensively analyzed using the ‘TCGAplot’ package in R, encompassing immune inhibitors, immune checkpoints, immune cell infiltrates, and immune scores. Visualization of the results was achieved through heatmaps and scatter plots generated using the ggplot2 package in R.

To obtain high-resolution insights into MYBL2 expression across immune cell subtypes, single-cell RNA sequencing (scRNA-seq) data analysis was conducted using the HCCDB web application, in which cell type identification, MYBL2 expression profiling, and differential expression analysis between various immune cell populations were conducted [23]. Visualization of scRNA-seq results was performed using UMAP (Uniform Manifold Approximation and Projection) plots to represent cell clusters and MYBL2 expression patterns.

### 2.7 Cell Culture

Human HCC cell line HepG2 was purchased from the American Type Culture Collection (ATCC, Manassas, VA, USA; Cat. No. ATCC HB-8065). To ensure cell line identity and prevent contamination, the cell line was authenticated via Short Tandem Repeat (STR) profiling and confirmed to be mycoplasma-free using standard detection methods. The cells were cultured in Dulbecco’s Modified Eagle Medium (DMEM; Thermo Fisher Scientific; Cat. No. 11965-092; Waltham, MA, USA) medium supplemented with 10% heat-inactivated fetal bovine serum (FBS; GIBCO, Thermo Fisher Scientific, Cat. No. 16140-071, Waltham, MA, USA), glutamax, and antibiotic–antimycotic at 37°C in a humidified incubator with 5% CO_2_. Cells were seeded at a density of 1.5 × 10^6^ cells per 6 cm culture dish. Twenty-four hours after seeding, we transfected the HepG2 cells with a concentration of 25 nM miR-29a mimic (Cat. No. C-310521-07-0002) (Dharmacon, GE Healthcare, CO, USA) or miR control (Cat. No. CN-001000-01) (Dharmacon, GE Healthcare (now Horizon Discovery), CO, USA) for 24 h with Lipofectamine™ RNAiMAX Transfection Reagent (Invitrogen, Thermo Fisher Scientific, Waltham, MA, USA; Cat. No. 13778150) according to the manufacturer’s instructions.

### 2.8 Western Blot

Cells or fresh frozen livers were homogenized using MagNA Lyser (Roche, Mannheim, Germany; Cat. No. 03 358 968 001) in PRO-PREP^™^ Protein Extraction Solution (iNtRON Biotechnology, Seongnam, Republic of Korea; Cat. No. 17081). Thirty micrograms of proteins were analyzed through sodium dodecyl sulfate–polyacrylamide gel electrophoresis (SDS-PAGE) and immunoblotting. The primary antibodies anti-MYBL2 (1:40,000, PROTEINTECH, IL, USA; Cat. No. 18896-1-AP) and anti-glyceraldehyde 3-phosphate dehydrogenase (GAPDH) (1:80,000, PROTEINTECH, IL, USA; Cat. No.10494-1-AP). For protein detection, blots were incubated with HRP-conjugated anti-rabbit immunoglobulin-G secondary antibodies (1:10,000; PerkinElmer, MA, USA; Cat. No. NEF812001EA). Chemiluminescent signals were developed using the Western Lightning Plus-ECL system (PerkinElmer, MA, USA; Cat. No. NEF812001EA) and subsequently quantified using ImageJ software.

### 2.9 Luciferase Activity Assay

The oligonucleotides that contained the MYBL2 3^′^UTR target sequence (5^′^-UGGUGCU-3^′^) or MYBL2-3^′^UTR-MUT (mutant) sequence (5^′^-UCCUCGU-3^′^) were annealed and cloned into the pMIR-REPORTTM miRNA Expression Reporter Vector (Ambion^™^, Thermo Fisher Scientific, Waltham, MA, USA; Cat. No. AM5795) to generate the pMIR-MYBL2-29a vector or pMIR-MYBL2-MUT vector (negative control). The plasmids were purified using the EasyPrep EndoFree Maxi Plasmid Extraction Kit (BIOTOOLS, Taipei, Taiwan; Cat. No. DPT-BA17). HepG2 cells were cultured in a 10 cm dish and were transfected with 6 μg DNA plasmids of pMIR-MYBL2-29a or pMIR-MYBL2-MUT using TurboFect Transfection reagent (Thermo Fisher Scientific, Waltham, MA, USA). After 24 h of transfection, cells were seeded on a 6 cm dish with a 1.0 × 10^6^ cells/dish density and were grown overnight. The cells were transfected with miR-29a mimic or miR control for 24 h using the RNAiMAX transfection reagent according to the manufacturer’s instructions. After 48 h of transfection, the luciferase activity was measured using Luciferase Reporter Assay Kits (neolite, PerkinElmer, Waltham, MA, USA; Cat. No. R0531).

### 2.10 Animal Study

All animal protocols were reviewed and approved by the Institutional Animal Care and Use Committee (IACUC) of Chang Gung Memorial Hospital (application number: #2020121109) and were conducted in accordance with the National Institutes of Health Guide for the Care and Use of Laboratory Animals. All animals were housed in a conventional facility at 22°C with a relative humidity of 55% in a 12 h light/12 h dark cycle with food and sterile tap water available *ad libitum*. Mice were purchased from BioLASCO Taiwan Co., Ltd. (Taipei, Taiwan). Nine-week-old male wild-type (WT) and miR-29a transgenic mice, with a C57BL/6 background, were utilized to investigate the role of miR-29a in HCC. To induce HCC, mice were subjected to a western diet (WD; Teklad diets, TD.120528, Envigo, Madison, WI, USA; Cat. No. TD.120528) and CCl_4_ (Sigma-Aldrich, 289116-100ML, St. Louis, MO, USA; Cat. No.: 289116-100ML) treatment for 25 weeks as described previously [32]. The WD consisted of 21.1% fat, 41% sucrose, and 1.25% cholesterol. Mice were intraperitoneally injected with CCl_4_ at a dose of 0.32 µg/gram body weight, once a week for 25 weeks [33]. The control mice were fed a normal diet (ND). The study included a total of 16 mice, equally distributed into four groups (N = 4 per group): WT-ND/CCl_4_, WT-WD/CCl_4_, miR-29aTg-ND/CCl_4_, and miR-29aTg-WD/CCl_4_. Sample size was determined using the Resource Equation Method for exploratory studies, adhering to the 3Rs principle of Reduction. Despite the small cohort size, the robust phenotypic differences observed provided sufficient statistical power to detect significant effects. The miR-29a transgenic mice, which overexpress miR-29a driven by the phosphoglycerate kinase 1 (PGK1) promoter, were generated and bred as previously described [34]. At the experimental endpoint, mice were deeply anesthetized with 40 mg/kg zoletil and 10 mg/kg xylazine. Maximal blood volume was obtained by cardiac puncture. Immediately after exsanguination, cervical dislocation was carried out to ensure euthanasia, in accordance with the recommendations of the National Institutes of Health Guide for the Care and Use of Laboratory Animals guidelines.

### 2.11 iTRAQ Gel-Free Proteomics

After transfection with the miR-29a mimic or miR control for 24 h, the cell pellets were lysed in protein extraction reagent (iNtRON Biotechnology, Seongnam, Republic of Korea; Cat. No. 17081), homogenized, and centrifugated. Protein extracts (100 μg) were prepared for a relative and absolute quantitation (iTRAQ) gel-free proteomics assay. The samples were first subjected to high-abundance protein depletion with the Pierce Top 12 Abundant Protein Depletion Spin Columns (Thermo Fisher Scientific, Waltham, MA, USA; Cat. No. 85165) and then sent to sample preparation with the iTRAQ Reagents Multiplex Kit (Sciex, Framingham, MA, USA; Cat. No. 4352135). After passing the standard quality control (QC) check, the labeled proteins were analyzed with LC/Q-Exactive Orbitrap MS (Thermo Fisher Scientific, Waltham, MA, USA) for 24 h, and the generated raw data were analyzed with Proteome Discoverer v2.4 (Thermo Fisher Scientific, Waltham, MA, USA) by referring to the MASCOT 2.5 database (Matrix science, London, UK).

### 2.12 Statistical Analysis, Software and Statistical Packages

Data are expressed as the mean ± standard deviation of at least three independent experiments. *p* < 0.05 was considered to indicate a statistically significant difference. For high-dimensional comparisons, including differential gene expression and pathway enrichment analyses, *p*-values were adjusted using the Benjamini-Hochberg False Discovery Rate (FDR) method, with a significance threshold of q < 0.05. Correlations between *MYBL2* expression and drug sensitivity (GDSC/CTRP databases) or immune infiltration estimates were considered exploratory hypothesis-generating analyses; nominal *p*-values are reported for these associations, though key findings (e.g., Sorafenib) remained significant after FDR adjustment. All statistical analyses were performed using R software (version 4.1.0). Key packages included ‘survival’ (v3.2-13) for Cox regression, ‘pROC’ (v1.18.0) for ROC analysis, and ‘Seurat’ (v4.0) for single-cell analysis. Nomogram construction was performed by using Nomogram COX results visualization tool in Hiplot Pro (https://hiplot.com.cn/), a comprehensive web service for biomedical data analysis and visualization. A complete list of software versions and database URLs is provided in Supplementary Table S1.

## 3 Results

### 3.1 Identification of MYBL2 as a Predictive Biomarker

**Figure 1:**
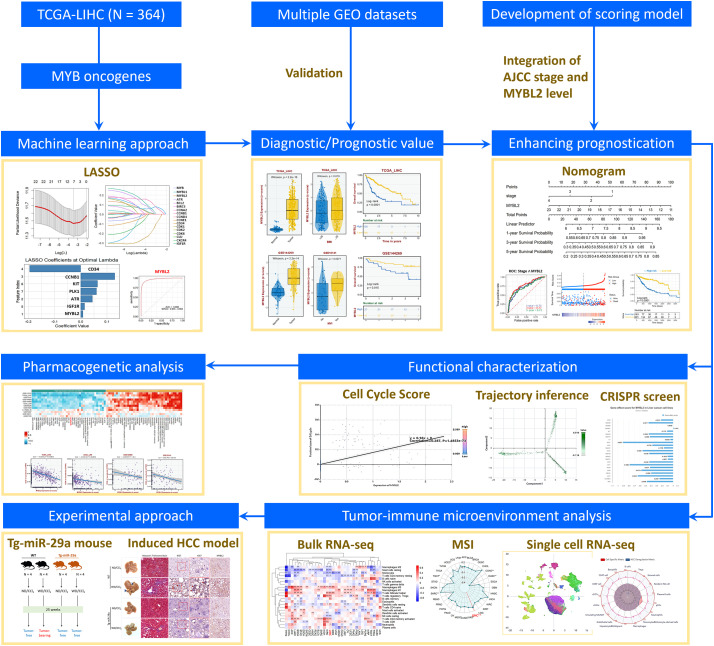
Flowchart of identifying the significance of MYBL2 in clinical prediction, molecular function, tumor microenvironment and possible therapy. *p < 0.05 **p < 0.01. Abbreviations: MYBL2, MYB proto-oncogene like 2; LIHC, Liver hepatocellular carcinoma; TCGA, The Cancer Genome Atlas; GEO, Gene Expression Omnibus; GTEx, Genotype-Tissue Expression; LASSO, Least Absolute Shrinkage and Selection Operator; AJCC, American Joint Committee on Cancer; ROC, Receiver Operating Characteristic; GSEA, Gene Set Enrichment Analysis; GDSC, Genomics of Drug Sensitivity in Cancer; CRISPR: Clustered Regularly Interspaced Short Palindromic Repeats; CTRP, Cancer Therapeutics Response Portal; PRISM, Profiling Relative Inhibition Simultaneously in Mixtures; MSI, Microsatellite Instability; HCC, Hepatocellular carcinoma; WD, western diet; CCl_4_, Carbon tetrachloride

**Figure 2:**
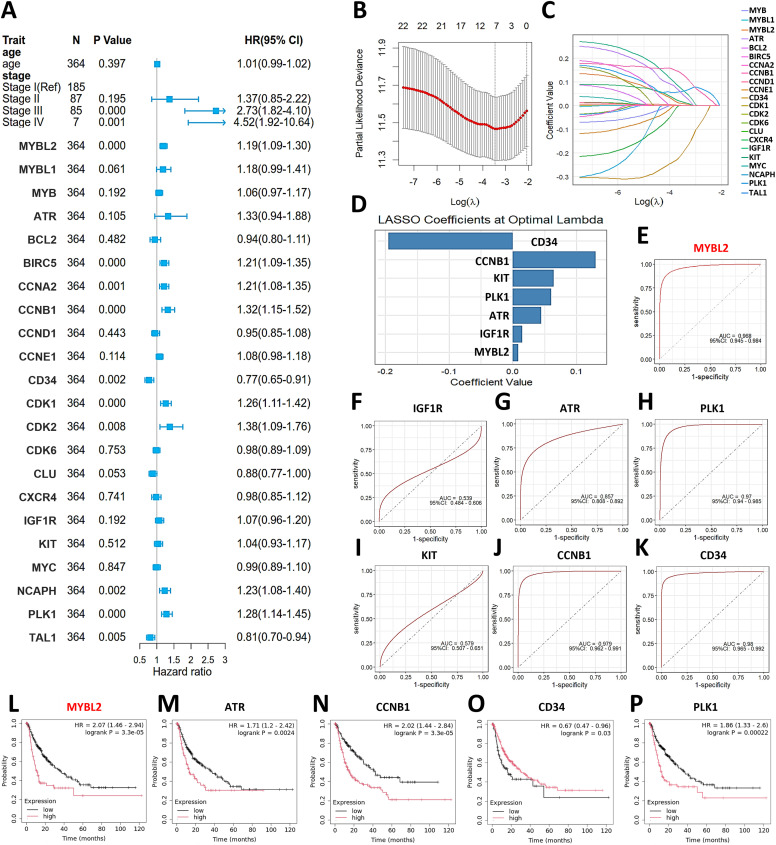
Identification of MYBL2 as most relevant biomarker for diagnosis and prognosis. (**A**) Forest plot exhibiting Cox hazard model that examines the effect of AJCC stage and MYB oncogenes and their downstream targets on the survival rate of the TCGA-LIHC cohort. HR, hazard ratio. (**B**) Dot plot showing the selection of the optimal regularization parameter (λ, lambda) for the LASSO model through 10-fold cross-validation. The optimal λ value was determined by identifying the point that minimizes cross-validation error while maintaining model parsimony. (**C**) Line chart visualizing the change of each gene coefficient as λ varies. Genes whose coefficients remain non-zero even at high λ values are considered more relevant. (**D**) Bar chart of the coefficient value. (**E–K**), ROC curve analyzing the discriminatory power between normal tissue and tumor tissue for MYBL2 (**E**), IGF1R (**F**), ATR (**G**), PLK1 (**H**), KIT(**I**), CCNB1 (**J**) and CD34 (**K**). (**L–P**), Kaplan-Meier plot analyzing relapse-free survival probability of the TCGA cohort stratified by low and high gene expression levels of MYBL2 (**L**), ATR (**M**), CCNB1 (**N**), CD34 (**O**) and PLK1(**P**). Abbreviations: HR, hazard ratio; AJCC, American Joint Committee on Cancer; ATR, Ataxia Telangiectasia and Rad3 related; BCL2, B-cell lymphoma 2; BIRC5, Baculoviral IAP repeat-containing protein 5; CCNA2, Cyclin A2; CCNB1, Cyclin B1; CCNE1, Cyclin E1; CD34, Cluster of differentiation 34; CDK1, Cyclin-dependent kinase 1; CDK2, Cyclin-dependent kinase 2; CDK6, Cyclin-dependent kinase 6; CLU, Clusterin; CXCR4, C-X-C motif chemokine receptor 4; IGF1R, Insulin-like growth factor 1 receptor; KIT, KIT proto-oncogene, receptor tyrosine kinase; KM (in KM plotter), Kaplan–Meier; LASSO, Least Absolute Shrinkage and Selection Operato; MYBL1: MYB proto-oncogene like 1; MYBL2: MYB proto-oncogene like 2; MYC, MYC proto-oncogene, bHLH transcription factor; NCAPH, Non-SMC condensin I complex subunit H; PLK1, Polo-like kinase 1; TAL1, T-cell acute lymphocytic leukemia protein 1

The flowchart for this study is illustrated in Fig. 1. First, we utilized the Cox Hazard Model to examine the effect of AJCC stage and MYB oncogenes and their downstream targets on the survival rate of the TCGA-LIHC cohort, revealing that stage III, stage IV and 10 genes including MYBL2, BIRC5, CCNA2, CCNB1, CD34, CDK1, CDK2, NCAPH, PLK1 and TAL1 showed significant association with survival (Fig. 2A). We utilized the machine learning algorithm LASSO to identify the most relevant genes for survival prediction. This approach determined the optimal regularization level (Fig. 2B), revealed genes that remained significant at this regularization level (Fig. 2C), and yielded coefficients for seven relevant genes (Fig. 2D): CD34, CCNB1, KIT, PLK1, ATR, IGF1R and MYBL2. Subsequently, we assessed these seven LASSO-selected genes for their ability to discriminate between normal and tumor tissue (Fig. 2F–K). Further investigation into the ability of these genes to stratify patients into high and low relapse-free survival groups (Fig. 2L–P) identified MYBL2 as the most predictive biomarker.

**Figure 3:**
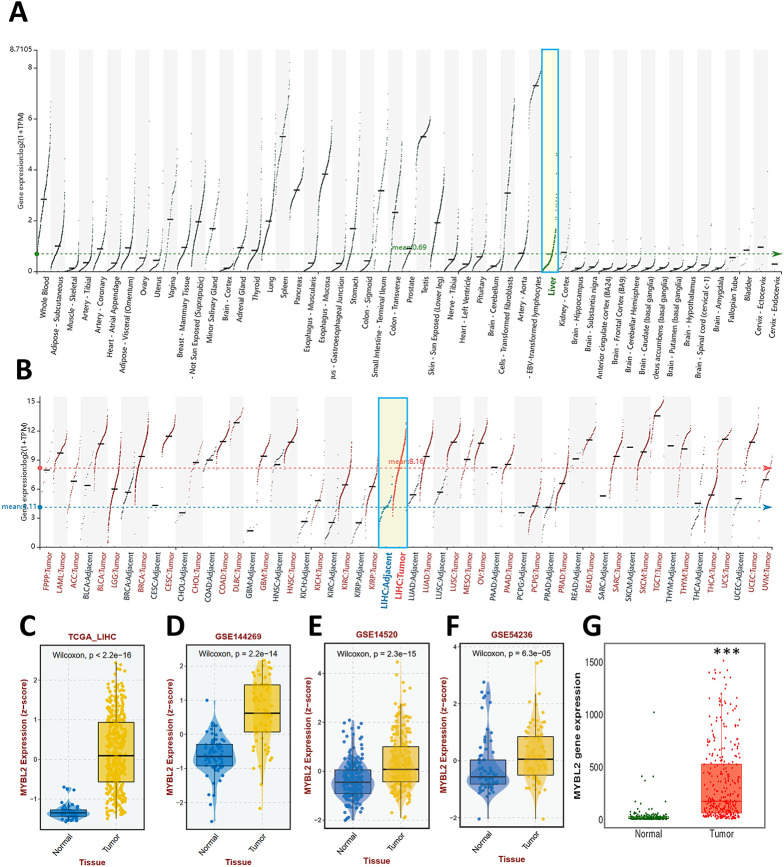

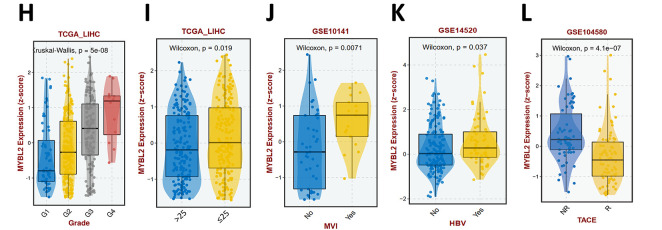
Differential expression profile of MYBL2. (**A**,**B**), MYBL2 expression levels across diverse normal tissue types (**A**) and various pan-cancer types (**B**). Green boxes highlight liver tissues. (**C–G**), Box plot depicting MYBL2 expression levels in normal tissue and tumor tissue in cohorts of TCGA-LIHC (**C**), GSE144269 (**D**), GSE14520 (**E**), GSE54236 (**F**) and TNMplot datasets (**G**). ***p < 0.001 when compared to normal tissue. (**H–L**), Box plots depicting MYBL2 expression levels in relation to tumor grades (**H**), patients with low and high BMI (**I**), the presence of MVI (**J**), HBV positivity (**K**), and non-response to TACE (**L**). Abbreviations: MYBL2, MYB proto-oncogene like 2; TCGA_LIHC, The Cancer Genome Atlas-Liver hepatocellular carcinoma; BMI, body mass index; MVI, microvascular invasion; HBV, hepatitis B virus; TACE, transarterial chemoembolization. FPPP, FFPE Pilot Phase II; LAML, acute myeloid leukemia; ACC, adrenocortical carcinoma; BLCA, bladder urothelial carcinoma; BRCA, breast invasive carcinoma; CESC, cervical squamous cell carcinoma and endocervical adenocarcinoma; CHOL, cholangiocarcinoma; COAD, colon adenocarcinoma; DLBC, lymphoid neoplasm diffuse large B-cell lymphoma; ESCA, esophageal carcinoma; GBM, glioblastoma multiforme; HNSC, head and neck squamous cell carcinoma; KICH, kidney chromophobe; KIRC, kidney renal clear cell carcinoma; KIRP, kidney renal papillary cell carcinoma; LGG, brain lower grade glioma; LIHC, liver hepatocellular carcinoma; LUAD, lung adenocarcinoma; LUSC, lung squamous cell carcinoma; MESO, mesothelioma; OV, ovarian serous cystadenocarcinoma; PAAD, pancreatic adenocarcinoma; PCPG, pheochromocytoma and paraganglioma; PRAD, prostate adenocarcinoma; READ, rectum adenocarcinoma; SARC, sarcoma; SKCM, skin cutaneous melanoma; STAD, stomach adenocarcinoma; TGCT, testicular germ cell tumor; THCA, thyroid carcinoma; THYM, thymoma; UCEC, uterine corpus endometrial carcinoma; UCS, uterine carcinosarcoma; UVM, uveal melanoma

Comprehensive analysis of gene expression indicated basal expression levels of hepatic MYBL2 across normal tissue (Fig. 3A). Elevated MYBL2 expression levels were noted across TCGA pan-cancer types, including HCC (Fig. 3B,C). The expression patterns of GSE144269 (Fig. 3D), GSE14520 (Fig. 3E), and GSE54236 (Fig. 3F) were in line with the TCGA cohort. TNMplot shows a similar pattern (Fig. 3G). Furthermore, high MYBL2 levels correlated significantly with conditions indicative of poor prognosis, including high tumor grade (Fig. 3H), low body mass index (BMI) (Fig. 3I), presence of microvascular invasion (MVI) (Fig. 3J), hepatitis B virus (HBV) positivity (Fig. 3K), and non-response to TACE (Fig. 3L).

### 3.2 MYBL2-Based Prognostic Scoring Model Enhances Clinical Prognostication

**Figure 4:**
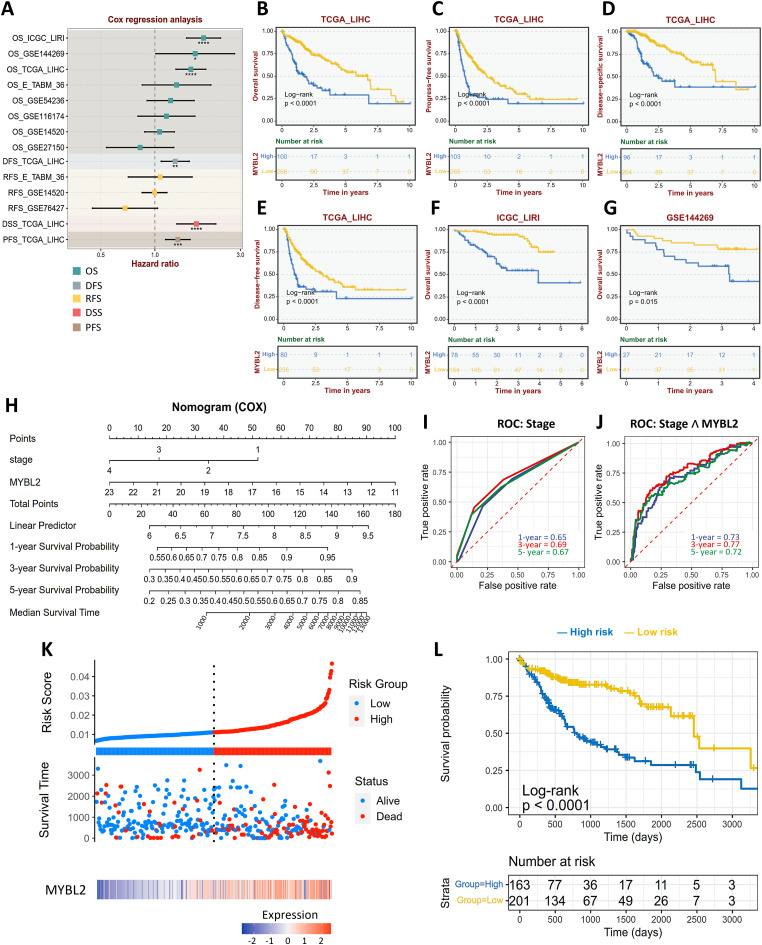
MYBL2-based Prognostic Scoring Model Enhances Clinical Prognostication. (**A**), Forest plot depicting Cox regression analysis of the effect of MYBL2 expression levels on the probability of OS, DFS, RFS, DSS, and PFS. *p < 0.05, **p < 0.01, ***p < 0.001, ****p < 0.0001. (**B–G**), Kaplan-Meier plot illustrating probability of OS (**B**), PFS (**C**), DSS (**D**) and DFS (**E**) in TCGA cohort, OS in International Cancer Genome Consortium—Liver Cancer—Riken, Japan (ICGC-LIRI) cohort (**F**) and OS in GSE144269 cohort (**G**) stratified by low and high MYBL2 expression levels. (**H**), Nomogram scoring model incorporating AJCC stage categories and MYBL2 expression levels for prognostic prediction. (**I**,**J**), ROC curve analyzing the prediction accuracy on survival for AJCC stage categories only (**I**) and for the prognostic scoring model incorporating AJCC stage categories with MYBL2 expression levels. (**K**), Risk score analysis depicting the impact of low and high score on status (alive or dead) and survival time. The dotted line represents the median risk score. (**L**), Kaplan-Meier pot illustrating OS probability stratified by low and high-risk score. Abbreviations: MYBL2, MYB proto-oncogene like 2; OS, overall survival; DFS, disease-free survival; RFS, relapse-free survival; DSS, disease-specific survival; PFS, progression-free survival; TCGA_LIHC, The Cancer Genome Atlas—Liver hepatocellular carcinoma; ICGC_LIRI, International Cancer Genome Consortium—Liver Cancer, Riken, Japan; GSE144269, Gene Expression Omnibus series 144269; AJCC, American Joint Committee on Cancer; ROC, receiver operating characteristic; AUC, area under the curve

A Cox regression analysis summarized the impact of MYBL2 expression on HCC cohorts, revealing that elevated levels are indicative of unfavorable outcomes (Fig. 4A), including in the TCGA cohort (Fig. 4B–E), the ICGC cohort (Fig. 4F) and the GSE144269 cohort (Fig. 4G). To develop a more comprehensive prognostic model, we created a nomogram integrating MYBL2 expression with AJCC stage, generating a prognostic score for each patient (Fig. 4H). Compared to predictions based on stage alone (AUC values of 0.65, 0.69, and 0.67 for 1-, 3-, and 5-year survival, respectively) (Fig. 4I), our prognostic model incorporating both stage and MYBL2 expression demonstrated superior predictive power (AUC values of 0.73, 0.77, and 0.72 for 1-, 3-, and 5-year survival, respectively) (Fig. 4J). Risk score analysis revealed that higher scores were associated with increased mortality and shorter survival times (Fig. 4K). Moreover, the prognostic score effectively stratified HCC patients into groups with significantly different survival probabilities (Fig. 4L). Collectively, these results demonstrate that combining MYBL2 expression with AJCC stage significantly enhances clinical prognostication.

### 3.3 Relationship of MYBL2 with Known Biomarkers and Biological Function

**Figure 5:**
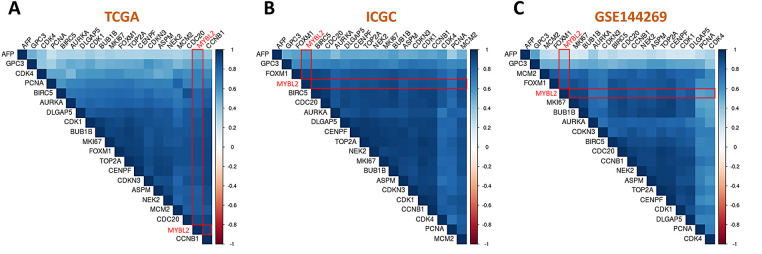

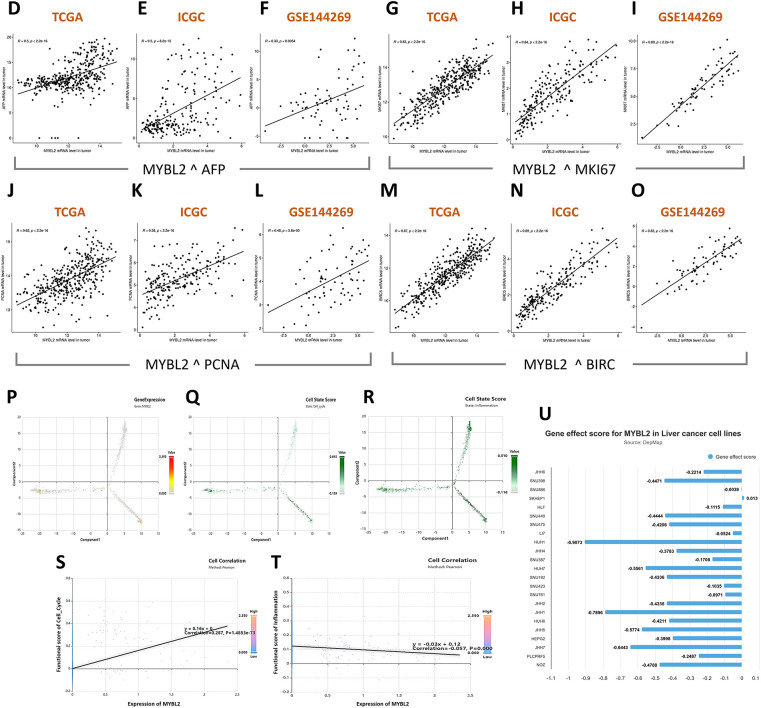
Association of MYBL2 with poor outcome indicators, tumor progression and immune signaling. (**A–C**), Matrix heatmaps representing correlation of MYBL2 with known poor outcome indicators for HCC cohort in TCGA (**A**), ICGC (**B**), and GSE144269 (**C**). Color key: Red indicates negative correlation, blue indicates positive correlation, with color intensity proportional to Pearson correlation coefficient strength (r values ranging from −1 to +1). White/neutral colors represent weak or no correlation. (**D–O**), Scatter plots illustrating correlation analysis of MYBL2 with AFP (**D**–**F**), with MKI67 (**G**–**I**), with PCNA (**J**–**L**), and with BIRC (**M**–**O**). (**P–T**), Single-cell transcriptomic analysis of GSE125449 dataset exploring the causative interplay between MYBL2 gene expression and cell states influencing cell fate determination. Monocle-based trajectory inference plot depicting MYBL2 gene expression (**P**) and cell fate score for Cell Cycle (**Q**) and for Inflammation (**R**). Pearson correlation results of the MYBL2 with Cell Cycle (**S**) and Inflammation (**T**). (**U**), Gene effect score derived from CRISPR screens sourced for DepMap. Negative scores imply cell growth inhibition following MYBL2 knockout. Scores are normalized such that nonessential genes have a median score of 0. MYBL2, Abbreviations: MYB proto-oncogene like 2; TCGA, The Cancer Genome Atlas; ICGC, International Cancer Genome Consortium; GSE144269, Gene Expression Omnibus series 144269; AFP, alpha-fetoprotein; GPC3, glypican-3; CDK4, cyclin-dependent kinase 4; PCNA, proliferating cell nuclear antigen; BIRC5, baculoviral IAP repeat-containing protein 5; AURKA, aurora kinase A; DLGAP5, discs large-associated protein 5; CDK1, cyclin-dependent kinase 1; MKI67, marker of proliferation Ki-67; FOXM1, forkhead box protein M1; TOP2A, DNA topoisomerase II alpha; CENPF, centromere protein F; CDKN3, cyclin-dependent kinase inhibitor 3; ASPM, abnormal spindle microtubule assembly; NEK2, NIMA-related kinase 2; MCM2, minichromosome maintenance complex component 2; CDC20, cell division cycle 20; CCNB1, cyclin B1; HCC, hepatocellular carcinoma; Cell Cycle, cell cycle state score; Inflammation, inflammation state score; DepMap, Cancer Dependency Map; CRISPR, clustered regularly interspaced short palindromic repeats

To assess clinical applicability, we examined the correlation between MYBL2 and a panel of established HCC biomarkers. MYBL2 exhibited a positive correlation with most biomarkers (Fig. 5A–C), including AFP (Fig. 5D–F), MKI67 (Fig. 5G–I), PCNA (Fig. 5J–L), and BIRC (Fig. 5M–O). The causative interplay between MYBL2 gene expression levels and cell states that contribute to cellular development trajectory and fate was analyzed with CellTracer at a single cell transcriptomics level (Fig. 5P–R), revealing that MYBL2 positively correlated with the functional score of the Cell Cycle (Fig. 5S) while negatively correlated with that of Inflammation (Fig. 5T). It is worth noting that this “Inflammation” score likely reflects acute inflammatory signaling, which is often suppressed in established tumors, contrasting with the chronic, immunosuppressive immune infiltration observed in our TME analysis. Finally, we explored the role of MYBL2 in HCC cell viability by analyzing CRISPR screens from the DepMap database. Among 23 HCC cell lines, 21 demonstrated growth inhibition following MYBL2 knockout (Fig. 5U), indicating that MYBL2 exerts a pro-survival effect in HCC. Collectively, these findings demonstrate the potential involvement of MYBL2 in promoting cancer cell proliferation and in inhibiting tumor immunoreactivity.

### 3.4 Pharmacogenetic Analysis

**Figure 6:**
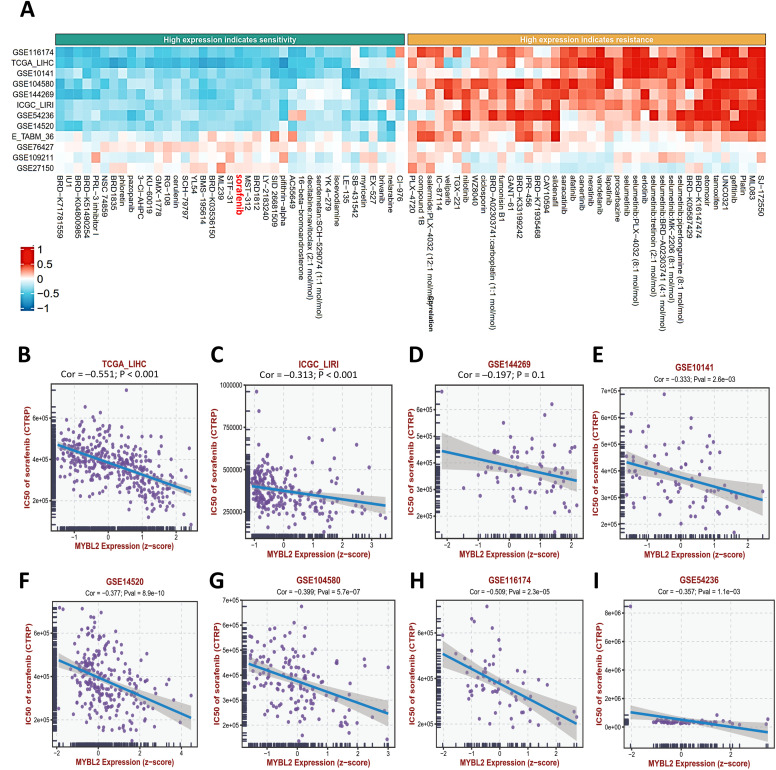
Impact of MYBL2 expression on response to targeted therapy. (**A**), Heatmap illustrating correlation of MYBL2 expression levels with sensitivity to various drugs across multiple HCC datasets. (**B–I**), Scatter plot exhibiting correlation of MYBL2 expression levels with IC50 value of sorafenib. Abbreviations: MYBL2, MYB proto-oncogene like 2; TCGA_LIHC, The Cancer Genome Atlas—Liver hepatocellular carcinoma; ICGC_LIRI, International Cancer Genome Consortium—Liver Cancer, Riken, Japan; CTRP, Cancer Therapeutics Response Portal; IC50, half-maximal inhibitory concentration

In a comprehensive multi-database pharmacogenetic survey (Fig. 6A), we observed that MYBL2 expression levels were negatively correlated with half-maximal inhibitory concentration (IC50) value of sorafenib in a variety of datasets (Fig. 6B–I).

### 3.5 Impact of MYBL2 on the MSI and an Immunosuppressive Tumor Microenvironment

**Figure 7:**
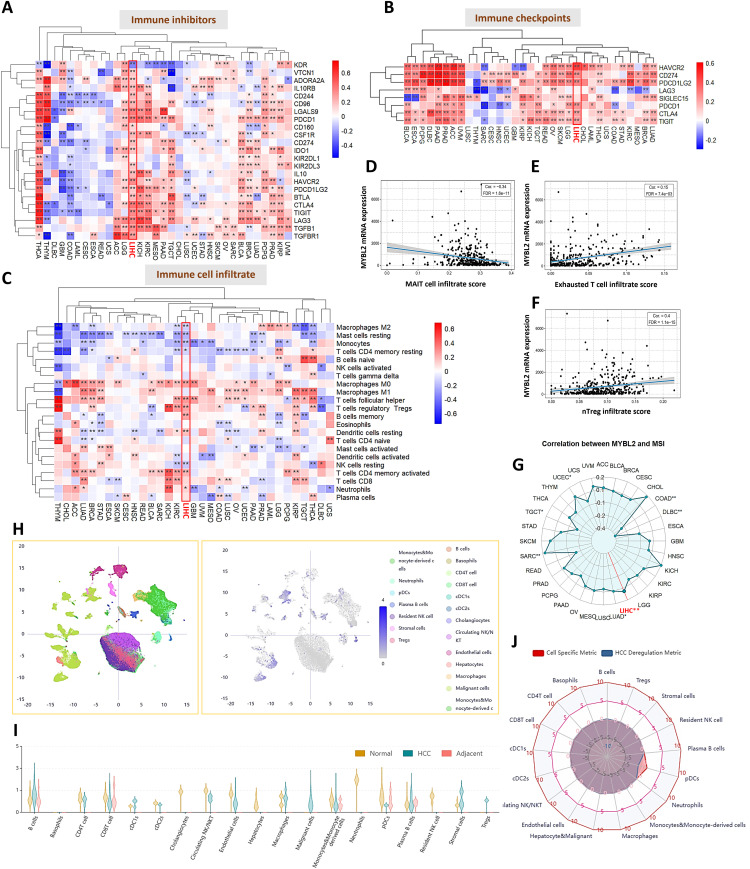
Impact of MYBL2 on an immunosuppressive tumor microenvironment. (**A–C**), Heatmap demonstrating Pearson’s correlation of MYBL2 gene expression levels with immune inhibitors (**A**), immune checkpoints (**B**), and immune cell infiltrate (**C**) across pan-cancer types. Liver hepatocellular carcinoma (LIHC) highlighted with red boxes. *p < 0.05 and **p < 0.01. (**D–F**), Scatter plot representing correlation of MYBL2 expression levels with score for mucosal-associated invariant T (MAIT) cells (**D**), exhausted T cells (**E**), and natural regulatory T cells (nTregs) (**F**). (**G**), Radar charts depicting Pearson’s correlation coefficients of MYBL2 expression levels with microsatellite instability (MSI). LIHC cohort highlighted in red. *p < 0.05 and **p < 0.01. (**H**), Uniform Manifold Approximation and Projection (UMAP) plot shows all cell subtypes deconvolution of HCDDB1 datasets comprising GSE192742 and GSE151530. (**I**), Violin plot showing MYBL2 expression levels of various cell sub-populations in normal tissue, HCC tissue, HCC adjacent normal tissue. (**J**), Summary radar chart illustrating MYBL2 expression levels across multiple cell sub-populations. Cell Specific Metric for fold change in log2 scale by comparing specific cell type with other cell type. HCC Deregulation Metric for fold change in log2 scale by comparing HCC with adjacent or normal cells in a specific cell type. Abbreviations: MYBL2, MYB proto-oncogene like 2; HCC, hepatocellular carcinoma; TCGA, The Cancer Genome Atlas; LIHC, liver hepatocellular carcinoma; MAIT, mucosal-associated invariant T (cell); nTregs, natural regulatory T cells; MSI, microsatellite instability; pDCs, plasmacytoid dendritic cells; UMAP, Uniform Manifold Approximation and Projection; HCDDB, hepatocellular carcinoma single-cell expression database; Tregs, regulatory T cells; PDCD1, programmed cell death protein 1 (PD-1); CD274, programmed death-ligand 1 (PD-L1); PDCD1LG2, programmed death-ligand 2 (PD-L2); CTLA4, cytotoxic T-lymphocyte–associated protein 4; HAVCR2, hepatitis A virus cellular receptor 2 (TIM-3); TIGIT, T cell immunoreceptor with Ig and ITIM domains

To clarify the impact of MYBL2 on tumor immune microenvironment, we examined the relationship between MYBL2 and immune signature. Bulk RNA-Seq analysis of the TCGA-LIHC cohort revealed that MYBL2 expression levels positively correlated with the majority of the immune inhibitors (Fig. 7A), as well as with genes of immune checkpoints (HAVCR2, CD274/PD-L1, PDCD1LG2/PD-L2, PDCD1/PD-1, CTLA4 and TIGIT) (Fig. 7B). While MYBL2 showed associations with some immunoreactive infiltrates (decreased macrophage M2 and monocyte; increased T follicular helper cells, memory B cells, and activated memory CD4 T cells), it also correlated with several potentially immunosuppressive infiltrates (macrophages M0, Tregs, resting dendritic cells, and neutrophils) (Fig. 7C). In addition, MYBL2 expression levels had a negative correlation with mucosal-associated invariant T (MAIT) cells (Fig. 7D), and a positive correlation with exhausted T cells (Fig. 7E), and natural regulatory T cells (nTregs) (Fig. 7F). Notably, MYBL2 expression levels positively correlated with the MSI (Fig. 7G), a predictive biomarker for immune checkpoint inhibitors. Furthermore, single-cell transcriptomic analysis (Fig. 7H) identified reduced MYBL2 expression levels in plasmacytoid dendritic cells (pDCs) of HCC compared to those of normal tissue and adjacent tissue (Fig. 7I,J). Taken together, these findings highlight MYBL2’s complex role in modulating the tumor immune microenvironment and its potential relevance to immune checkpoint therapy responses.

### 3.6 miR-29a Overexpression Inhibits HCC Tumorigenesis by Targeting MYBL2

**Figure 8:**
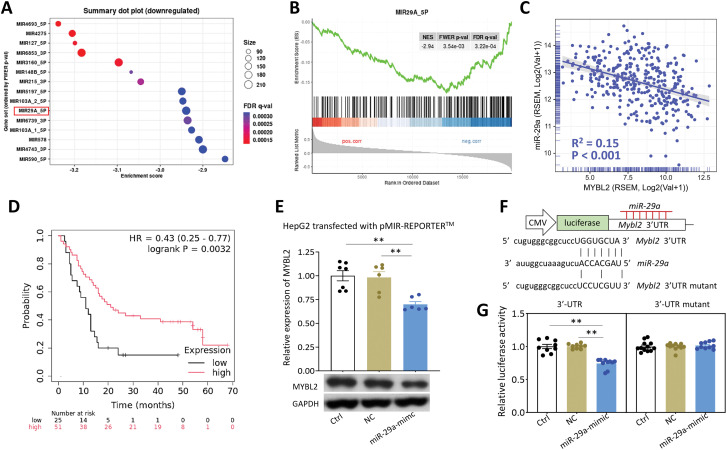

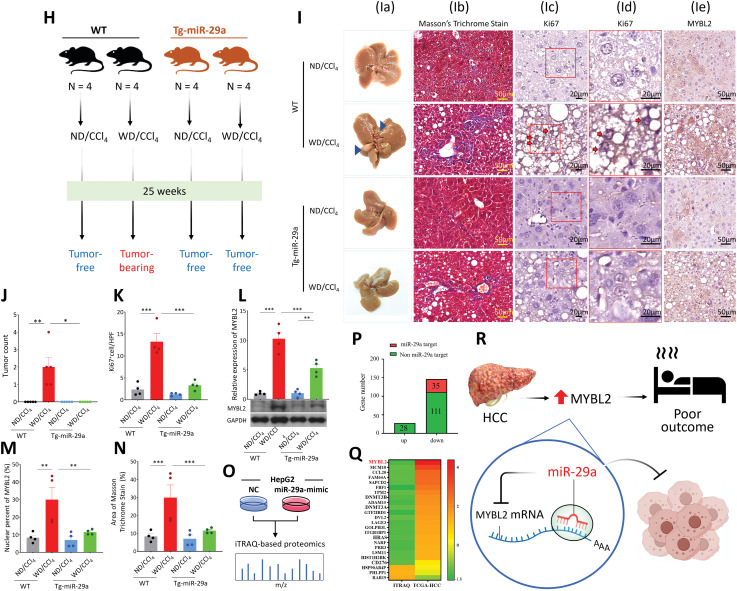
miR-29a overexpression inhibits HCC tumorigenesis by targeting MYBL2. (**A**), Dot plot summarizing significantly downregulated gene sets associated with MIRDB microRNA targets. (**B**), GESA plot showing normalized enrichment score (NES), family-wise error rate (FWER), false discovery rate (FDR). (**C**), Scatter plot representing Pearson’s correlation analysis of MYBL2 with miR-29a expression. (**D**), Kaplan-Meier plot depicting overall survival probability of HCC cohort from CapitalBio miRNA Array stratified by low and high miR-29a expression. HR, hazard ratio; *p*-value indicates statistical significance of survival difference between groups using log-rank test. (**E**), Bar chart exhibiting MYBL2 protein expression levels in control HepG2 cells with no transfection (Ctrl), with negative control sequence (NC) transfection and with miR-29a-mimic transfection. GAPDH as loading control. **p < 0.01 between indicated groups. (**F**), Schematic representation of the pMIR-REPORTER™ plasmid used in the CMV-driven luciferase assay to assess miR-29a interaction with MYBL2. (**G**), Bar chart exhibiting relative luciferase activity. **p < 0.01 between indicated groups. (**H**), Flowchart of a WD/CCl_4_-induced HCC model. (**I**), Representative images of livers excised from experimental mice (Ia). Masson’s Trichrome staining of liver sections (Ib). Immunohistochemical (IHC) staining for Ki67 protein expression (Ic), with red boxes indicating magnified regions (Id). IHC staining for MYBL2 protein expression (Ie). (**J–N**), Bar chart representing tumor count (**J**), Ki67 protein expression per high power field (HPF) (**K**), MYBL2 protein expression relative to GAPDH (**L**), nuclear percentage of MYBL2 (**M**) and percentage area of Masson’s Trichrome staining (**N**). *p < 0.05, **p < 0.01 and ***p < 0.001 between indicated groups. (**O**), Workflow diagram illustrating the iTRAQ-based proteomic analysis comparing HepG2 cells transfected with a negative control sequence (NC) and miR-29a mimic. (**P**), Bar chart showing the number of up-regulated and down-regulated genes identified through iTRAQ analysis. (**Q**), Heatmap displaying the top-ranked genes identified through iTRAQ analysis and validated in the TCGA cohort. The color intensity represents the relative expression levels of each gene across samples. (**R**), Proposed scheme depicting MYBL2 as a prognostic biomarker and potential target for miR-29a-mediated anti-tumor therapy. Abbreviations: miR-29a, microRNA-29a; HCC, hepatocellular carcinoma; MYBL2, MYB proto-oncogene like 2; MIRDB, microRNA target prediction database; GSEA, Gene Set Enrichment Analysis; NES, normalized enrichment score; FWER, family-wise error rate; FDR, false discovery rate; HR, hazard ratio; Ctrl, control; NC, negative control; GAPDH, glyceraldehyde-3-phosphate dehydrogenase; CMV, cytomegalovirus; 3^′^-UTR, 3^′^-untranslated region; WT, wild type; Tg-miR-29a, miR-29a transgenic; WD, western diet; CCl_4_, carbon tetrachloride; ND, normal diet; IHC, immunohistochemistry; HPF, high-power field; iTRAQ, isobaric tags for relative and absolute quantitation; TCGA, The Cancer Genome Atlas; OS, overall survival; mRNA, messenger RNA

To elucidate the regulatory mechanisms of MYBL2 and explore potential therapeutic approaches, we conducted MIRDB-based GSEA, which identified miR-29a as a putative negative regulator of MYBL2 (Fig. 8A,B). We subsequently confirmed a significant negative correlation between miR-29a and MYBL2 expression levels in the TCGA cohort (Fig. 8C). Notably, elevated miR-29a expression was associated with favorable overall survival outcomes in HCC patients (Fig. 8D).

Our *in vitro* study verified that miR-29a overexpression inhibited MYBL2 expression (Fig. 8E) through direct binding to the MYBL2 3^′^-UTR (Fig. 8F,G). To investigate the role of miR-29a overexpression in tumorigenesis *in vivo*, we utilized wild type (WT) mice and transgenic miR-29a (Tg) mice subjected to a WD/CCl_4_-induced HCC model (Fig. 8H). Compared to WT-WD/CCl_4_ mice exhibiting hepatic tumor formation, Tg-WD/CCl_4_ mice showed no tumor development, decreased expression levels of KI67 (a marker for cancer proliferation), reduced MYBL2 expression, and diminished nuclear percentage of MYBL2 (Fig. 8I–M). Furthermore, Tg-WD/CCl_4_ mice demonstrated reduced Masson’s Trichrome staining compared to WT-WD/CCl_4_ (Fig. 8N), suggesting that miR-29a overexpression confers a protective effect against WD/CCl_4_-induced liver fibrosis.

To profile the functional effect of miR-29a overexpression in HCC cells, we performed iTRAQ-based proteomics analysis (Fig. 8O). Among the downregulated genes, 35 genes were bioinformatically recognized as miR-29a targets (Fig. 8P), with MYBL2 emerging as the most significantly downregulated gene (Fig. 8Q). Pathway enrichment analysis of this downregulated cluster revealed a significant enrichment in “One-carbon metabolism” and “Cysteine” and methionine metabolism” pathways (Supplementary Fig. S1), reinforcing the functional consequence of the miR-29a-MYBL2 axis. Collectively, these findings establish MYBL2 as a prognostic biomarker for HCC and demonstrate its potential as an effective target of miR-29a in suppressing HCC development (Fig. 8R).

## 4 Discussion

Our comprehensive study, utilizing machine learning and experimental approaches, has identified MYBL2 as a promising prognostic biomarker and potential therapeutic target in HCC. The findings provide valuable insights into the role of MYBL2 in HCC pathogenesis and its potential as a clinical tool for patient stratification and treatment decision-making.

The identification of MYBL2 as a key prognostic marker through machine learning algorithms underscores the power of computational approaches in biomarker discovery. Our LASSO-based feature selection method effectively narrowed down a large set of candidate genes to identify MYBL2 as the most predictive biomarker for HCC. This outcome aligns with the growing utility of machine learning in genomic medicine [35–38]. Furthermore, our approach parallels recent ensemble learning frameworks developed for peptide classification [39–42], confirming that integrating computational feature selection with experimental validation is a robust strategy for identifying high-confidence therapeutic targets. This finding aligns with recent trends in utilizing machine learning for cancer biomarker discovery, as demonstrated in studies across various cancer types [43–45]. Notably, unlike previous reports focusing solely on MYBL2 as a proliferation marker in gastrointestinal (GI) cancers, our study uniquely identifies a miR-29a-MYBL2 axis specifically modulated by metabolic stress (western diet) and establishes a novel link to sorafenib sensitivity, which is distinct from its roles in other malignancies.

The elevated expression of MYBL2 in HCC tissues compared to normal liver tissue, and its association with poor prognostic factors such as high tumor grade, microvascular invasion, and HBV positivity, highlights its potential as a robust biomarker. These findings are consistent with recent studies that have implicated MYBL2 in the progression of other cancer types. For instance, prior research by Ren et al. reported similar associations between MYBL2 expression and poor prognosis in colorectal cancer [46].

Our development of a nomogram-based prognostic model integrating MYBL2 expression with AJCC stage represents a significant advancement in HCC prognostication. The improved predictive accuracy of this model compared to AJCC stage alone demonstrates the added value of molecular markers in refining clinical risk stratification. This methodology is concordant with the growing trend of incorporating molecular data into clinical decision-making [47–50].

The immune landscape analysis revealed a complex “inflamed but immunosuppressed” microenvironment. While high MYBL2 expression correlates with increased infiltration of cytotoxic cells and a high MSI status—typically signs of an “immune-hot” tumor—the concurrent elevation of immune checkpoints (PD-L1, CTLA4), Tregs, and exhausted T-cell signatures suggests that these effectors are functionally impaired. This creates a net immunosuppressive niche that facilitates tumor escape despite high immunogenicity. Furthermore, the counterintuitive downregulation of MYBL2 in plasmacytoid dendritic cells (pDCs) may represent a distinct mechanism of immune evasion, potentially impairing pDC maturation or interferon production, though this specific cell-type effect requires further mechanistic validation. It is important to note that the link between MYBL2 and this immunosuppressive microenvironment remains correlational, based on bulk and single-cell transcriptomics. Whether MYBL2 directly regulates the secretion of chemokines that recruit Tregs or exhaust T cells has yet to be determined. Future studies utilizing co-culture systems of MYBL2-overexpressing HCC cells and autologous T cells are needed to establish a causal mechanism.

The positive correlation between MYBL2 and established HCC biomarkers such as AFP, MKI67, PCNA, and BIRC further validates its biological relevance in HCC. These associations suggest that MYBL2 may be involved in key oncogenic processes such as cell proliferation and apoptosis resistance. Our findings from the DepMap CRISPR screen, showing that MYBL2 knockout inhibits HCC cell growth, provide further evidence for its pro-survival role. These results are consistent with recent studies on MYBL2’s function in other cancers, such as the work by Chen et al. in breast cancer [18,44,51–53].

Regarding the therapeutic response, pharmacogenomic analysis revealed a robust correlation between high MYBL2 expression and lower IC50 values for sorafenib across multiple datasets. However, we acknowledge that these findings are based on *in vitro* cell line data. While this suggests MYBL2 levels might stratify patients for sorafenib response, this association remains observational and requires prospective clinical validation.

It is important to acknowledge that while our bioinformatic analysis encompassed diverse HCC etiologies (viral, alcohol, metabolic), our *in vivo* validation utilized a WD/CCl_4_ model, which specifically mimics metabolic-associated steatohepatitis (MASH)-driven HCC. This suggests that the miR-29a-MYBL2 regulatory axis may be particularly relevant in the context of metabolic liver disease and fibrosis. While this aligns with the growing global burden of metabolic HCC, future studies utilizing viral hepatitis models are needed to confirm if this therapeutic axis remains equally potent across all HCC etiologies.

Our study provides strong evidence for the miR-29a-MYBL2 regulatory axis through direct 3^′^-UTR binding assays, protein-level downregulation, and inverse correlation in transgenic mice. A limitation of the current study is the lack of rescue experiments (e.g., re-expressing MYBL2 in miR-29a-overexpressing cells) to definitively isolate MYBL2’s contribution from other miR-29a targets. While our iTRAQ proteomics identified MYBL2 as the top downregulated target, miR-29a likely regulates a network of oncogenes. Future studies utilizing specific rescue assays will be essential to dissect the precise extent to which MYBL2 suppression mediates the potent anti-tumor phenotype observed. Regarding the luciferase assay, while we demonstrated specificity using a mutant 3^′^-UTR control, future experiments should include a known miR-29a target as a positive control to strictly quantify comparative binding efficiency.

Rather than viewing these as isolated events, we propose an integrated model where MYBL2 acts as a central driver of a “high-proliferation, immune-exhausted” phenotype. Its primary role in accelerating the cell cycle (evidenced by correlations with MKI67/PCNA) likely creates a state of high replicative stress. This hyper-proliferative state may explain the paradoxical sensitivity to sorafenib, as rapidly dividing cells are often more dependent on the RAS/RAF/MEK pathways targeted by the drug [54–56]. Thus, while these tumors are biologically aggressive, their high-proliferative state creates a specific vulnerability to kinase inhibitors. Simultaneously, this high tumor burden and potential neoantigen load (suggested by the correlation with MSI) likely recruit immune infiltrates, which, under chronic stimulation, progress to an exhausted and suppressive state (Tregs, exhausted T cells) as observed in our immune profiling.

The identification of miR-29a as a negative regulator of MYBL2 opens up new avenues for therapeutic interventions. Our *in vitro* and *in vivo* experiments demonstrating the tumor-suppressive effects of miR-29a overexpression through MYBL2 inhibition provide a strong rationale for developing miRNA-based therapies targeting MYBL2 in HCC. This approach aligns with the growing interest in miRNA therapeutics in cancer, as evidenced by recent clinical trials [57–61].

Several limitations of this study warrant mention. First, while our LASSO-derived signature was validated across multiple transcriptomic datasets (ICGC, GEO), these are retrospective cohorts. The lack of an independent prospective validation cohort limits the immediate clinical generalizability of the findings. Second, while the addition of MYBL2 to the AJCC staging system improved the 5-year AUC from 0.67 to 0.72, we acknowledge this statistical improvement is modest. Future studies incorporating decision curve analysis (DCA) and net reclassification improvement (NRI) metrics in prospective settings are needed to definitively establish its clinical utility over standard staging alone. Third, our *in vitro* mechanistic validation relied primarily on the HepG2 cell line. While HepG2 is a widely used model, it does not fully capture the heterogeneity of adult HCC. Future studies should employ a broader panel of HCC cell lines (e.g., Huh7, Hep3B) and patient-derived organoids to confirm these findings. Similarly, while our pharmacogenomic analysis revealed a robust correlation between MYBL2 expression and sorafenib sensitivity, this remains an *in silico* prediction. Direct *in vitro* drug sensitivity assays are required to definitively validate MYBL2 as a predictive biomarker for sorafenib response. Finally, regarding our *in vivo* model, we utilized constitutive miR-29a transgenic mice. While this model robustly demonstrates the tumor-suppressive potential of the miR-29a-MYBL2 axis, it represents a developmental overexpression scenario rather than a therapeutic intervention. The complete absence of tumors in the transgenic group, while striking, may reflect systemic developmental effects beyond MYBL2 regulation. Inducible models or therapeutic delivery of miR-29a mimics in established tumors would be required to fully validate the translational potential suggested here. While Lin et al. have provided evidence for the therapeutic potential of miR-29a mimic delivery in breast cancer models [62], validation using inducible systems or treatment of established tumors would be essential to distinguish acute MYBL2-mediated tumor suppression from the developmental phenotypes observed in our constitutive model.

## 5 Conclusion

In conclusion, our study establishes MYBL2 as a multifaceted biomarker in HCC with significant implications for diagnosis, prognosis, and treatment response prediction. The integration of MYBL2 into clinical decision-making holds the potential to improve patient stratification and guide personalized treatment strategies. Furthermore, the elucidation of the miR-29a-MYBL2 regulatory axis provides a mechanistic foundation for developing novel therapeutic approaches. Future studies should focus on validating these findings in larger, prospective cohorts and further exploring the mechanistic details of MYBL2’s role in HCC pathogenesis and immune modulation. Additionally, the development and preclinical testing of miR-29a-based therapies targeting MYBL2 represent promising directions for translational research in HCC.

## Supplementary Materials





## Data Availability

The data that support the findings of this study are available from the Corresponding Author, [Hung-Yu Lin], upon reasonable request.

## Declaration of Generative Al in Scientific Writing

During the preparation of this work the authors used Gemini-2.5-Pro in order to proofread grammatical errors. After using this tool, the authors reviewed and edited the content as needed and take full responsibility for the content of the publication.

## Supplementary Materials

The supplementary material is available online at https://www.techscience.com/doi/10.32604/or.2025.075284/s1.
