# Supplementary material for: Integrative Machine Learning and Experimental Validation Identify MYBL2 as a Prognostic Biomarker and Therapeutic Target in Hepatocellular Carcinoma
Source: Oncol Res. 2026 Apr 22;34(5):26. doi: 10.32604/or.2026.075284 (PMC13126373; doi:10.32604/or.2026.075284)
Supplement: Supplementary file 1 [file OncolRes-34-75284-s001.docx]

**Supplementary Materials**

**Supplementary Table S1. Software versions and database URLs used in this study**

| **Software/Database** | **Version** | **URL** |
| --- | --- | --- |
| R | 4.5.2 | [**https://www.r-project.org/**](https://www.r-project.org/) |
| Bioconductor | 3.22 | [**https://www.bioconductor.org/**](https://www.bioconductor.org/) |
| sva | 3.56.0 | [**https://bioconductor.org/packages/sva**](https://bioconductor.org/packages/sva) |
| mice | 3.19.0 | [**https://cran.r-project.org/package=mice**](https://cran.r-project.org/package=mice) |
| glmnet | 4.1-10 | [**https://cran.r-project.org/package=glmnet**](https://cran.r-project.org/package=glmnet) |
| pROC | 1.18.5 | [**https://cran.r-project.org/package=pROC**](https://cran.r-project.org/package=pROC) |
| survival | 3.8-3 | [**https://cran.r-project.org/package=survival**](https://cran.r-project.org/package=survival) |
| survminer | 0.5.1 | [**https://cran.r-project.org/package=survminer**](https://cran.r-project.org/package=survminer) |
| rms | 8.1-0 | [**https://cran.r-project.org/package=rms**](https://cran.r-project.org/package=rms) |
| ggplot2 | 4.0.1 | [**https://cran.r-project.org/package=ggplot2**](https://cran.r-project.org/package=ggplot2) |
| Seurat | 5.4.0 | [**https://cran.r-project.org/package=Seurat**](https://cran.r-project.org/package=Seurat) |
| TCGA (UCSC Xena) | v36.0 | [**https://xena.ucsc.edu**](https://xena.ucsc.edu/) |
| GEO | – | [**https://www.ncbi.nlm.nih.gov/geo/**](https://www.ncbi.nlm.nih.gov/geo/) |
| GTEx Portal | – | [**https://gtexportal.org**](https://gtexportal.org/) |
| HCCDB | – | [**http://lifeome.net/database/hccdb/**](http://lifeome.net/database/hccdb/) |
| BEST | – | [**https://rookieutopia.com/BEST/**](https://rookieutopia.com/BEST/) |
| TNMplot | – | [**https://tnmplot.com**](https://tnmplot.com/) |
| KM Plotter | – | [**https://kmplot.com**](https://kmplot.com/) |
| IHGA | – | [**https://ihga.cancer-pku.cn/**](https://ihga.cancer-pku.cn/) |
| DepMap | – | [**https://depmap.org/portal/**](https://depmap.org/portal/) |
| GENI | – | [**https://geni.heilbrunn-lab.org/**](https://geni.heilbrunn-lab.org/) |
| CellTracer | – | [**http://www.regenmedlab.org/celltracer/**](http://www.regenmedlab.org/celltracer/) |
| GDSC | – | [**https://www.cancerrxgene.org/**](https://www.cancerrxgene.org/) |
| CTRP | – | [**https://portals.broadinstitute.org/ctrp/**](https://portals.broadinstitute.org/ctrp/) |
| PRISM | – | [**https://depmap.org/portal/prism/**](https://depmap.org/portal/prism/) |
| TCGAplot | 4.0.0 | [**https://github.com/tjhwangxiong/TCGAplot**](https://github.com/tjhwangxiong/TCGAplot) |
| Hiplot Pro | – | [**https://hiplot.com.cn**](https://hiplot.com.cn/) |


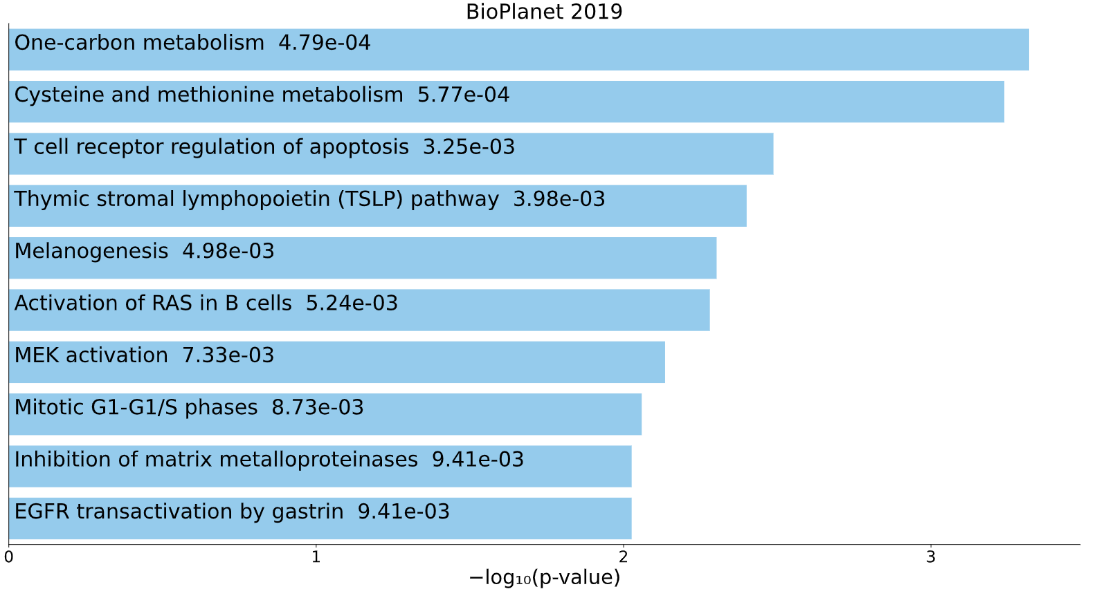


**Supplementary Figure S1. Pathway enrichment analysis of differentially expressed genes.**
To identify biological processes associated with the altered gene signature, we performed an enrichment analysis using the Enrichr web platform. The list of differentially expressed genes was inputted into Enrichr, and the BioPlanet 2019 database was selected to query for significantly enriched pathways. The bar chart ranks the top 10 enriched pathways based on the negative logarithm of their *p*-values, with longer bars indicating higher statistical significance. "One-carbon metabolism" and "Cysteine and methionine metabolism" were identified as the most significant metabolic alterations.
