# Supplementary figures and images for: Integrative Machine Learning and Experimental Validation Identify MYBL2 as a Prognostic Biomarker and Therapeutic Target in Hepatocellular Carcinoma
Source: Oncol Res. 2026 Apr 22;34(5):26. doi: 10.32604/or.2026.075284 (PMC13126373; doi:10.32604/or.2026.075284)

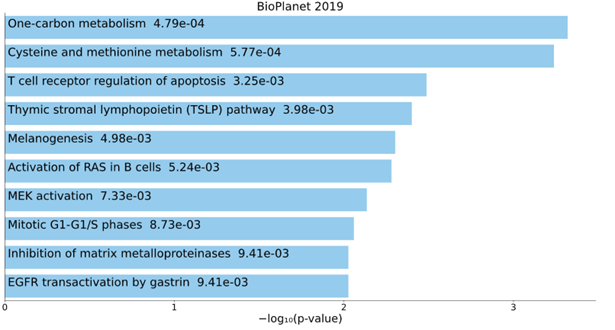

Supplement: Supplementary file 2 [file OncolRes-34-75284-s002.tif]
